# Application and prospects of single-cell and spatial omics technologies in woody plants

**DOI:** 10.48130/FR-2023-0027

**Published:** 2023-11-21

**Authors:** Shaoming Liang, Yiling Li, Yang Chen, Heng Huang, Ran Zhou, Tao Ma

**Affiliations:** 1 Key Laboratory of Bio-Resource and Eco-Environment of Ministry of Education, Sichuan Zoige Alpine Wetland Ecosystem National Observation and Research Station, College of Life Sciences, Sichuan University, Chengdu, China; 2 School of Forestry and Natural Resources, University of Georgia, Athens, GA, USA

**Keywords:** Woody plants, Single-cell transcriptomics, Single-cell epigenetics, Spatial transcriptomics

## Abstract

Over the past decade, high-throughput sequencing and high-resolution single-cell transcriptome sequencing technologies have undergone rapid development, leading to significant breakthroughs. Traditional molecular biology methods are limited in their ability to unravel cellular-level heterogeneity within woody plant tissues. Consequently, techniques such as single-cell transcriptomics, single-cell epigenetics, and spatial transcriptomics are rapidly gaining popularity in the study of woody plants. In this review, we provide a comprehensive overview of the development of these technologies, with a focus on their applications and the challenges they present in single-cell transcriptome research in woody plants. In particular, we delve into the similarities and differences among the results of current studies and analyze the reasons behind these differences. Furthermore, we put forth potential solutions to overcome the challenges encountered in single-cell transcriptome applications in woody plants. Finally, we discuss the application directions of these techniques to address key challenges in woody plant research in the future.

## Introduction

The common feature of multicellular organisms in nature is that they are comprised of tissues, organs, and systematic developmental programs that are coordinated and assembled by various cell types according to certain functional rules. In animals, more than 100 cell types, and even more cell subtypes, have been reported^[1−3]^. However, the different cell types and their heterogeneity in plants are still under investigation. Historically, the identification of plant cell types has mainly relied on the examination of cell morphology and the study of certain molecular markers identified in model plants such as *Arabidopsis*. Therefore, our understanding of the various cell types in plants, as well as the important scientific questions such as the heterogeneity among cell types, the composition of cell subtypes, the genetic developmental relationships, and the fate determination processes, is still limited, especially for woody plants characterized by highly lignified root and stem tissues.

Woody plants, as important components of terrestrial ecosystems, have played key roles in human history, both from socio-economic and subsistence standpoints. They fulfill essential ecological functions, including oxygen production, soil and water conservation, and climate regulation, in addition to being valuable sources of timber and wood^[4]^. It is the world's most abundant renewable resource used for timber, pulp, and energy^[5]^. An understanding of wood development^[6−9]^, environmental adaptability^[10−13]^, phenology^[14−17]^ and sex differentiation^[18−21]^ of woody plants has attracted significant attention. With the development of molecular biology and sequencing techniques, these studies have transitioned from using traditional quantitative polymerase chain reaction to characterize individual gene expression to analyzing the regulation of co-expressed genes using transcriptome data. However, transcriptome data alone cannot reveal the heterogeneity in gene expression between cells. As a result, our understanding of woody plants at the cellular level, including various cell types and molecular markers, remains poor.

**Figure 1:**
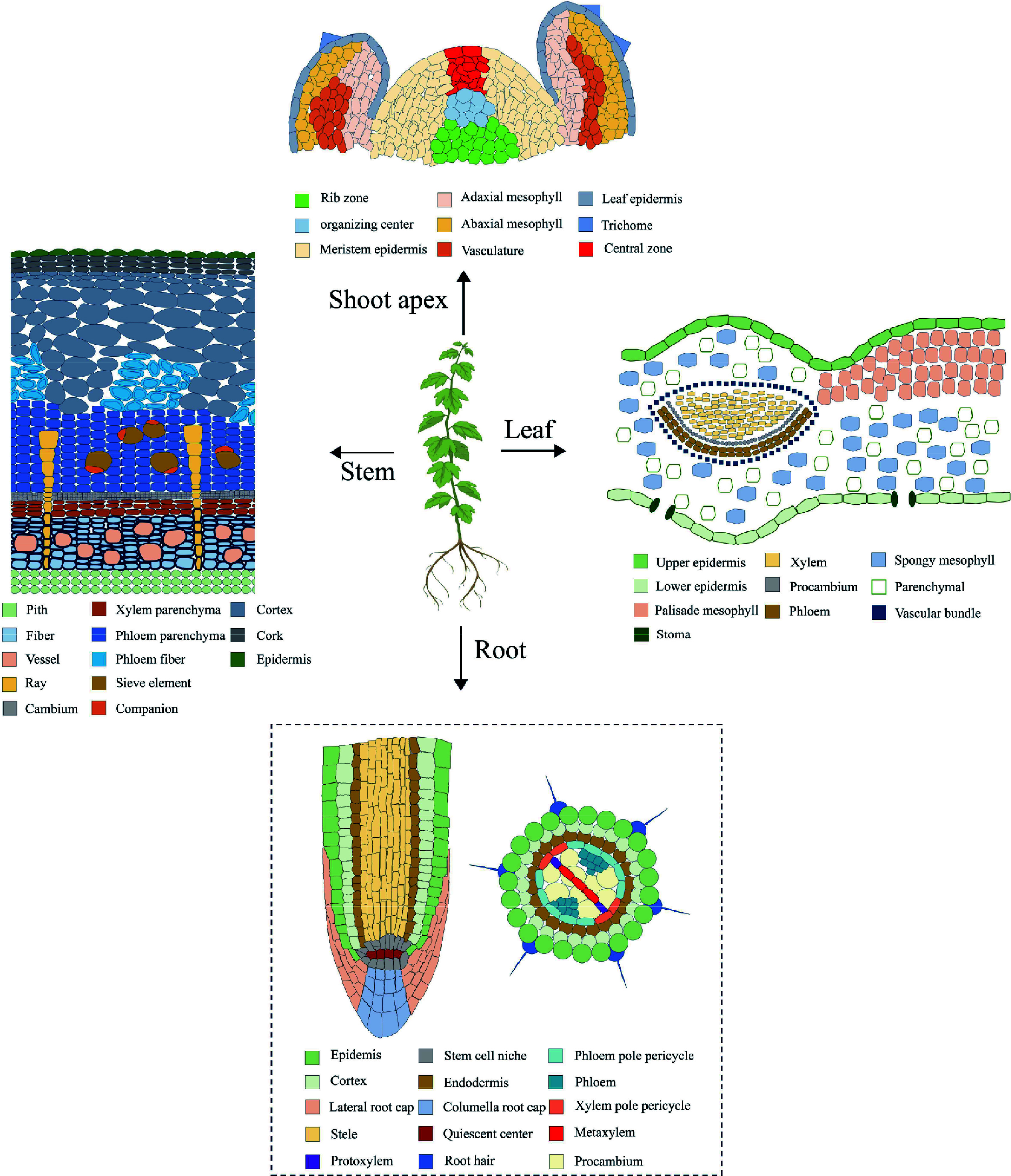
Cell types of roots, stems, leaves, and shoot apexes. The dotted lines indicate the absence of reported studies in woody plants.

Single-cell RNA sequencing (scRNA-seq) is a revolutionary technology capable of providing transcript-wide information at the level of individual cells, thereby enabling the elucidation of cellular heterogeneity and the identification of novel molecular markers. The technique has been applied to the study of various plants, including *Arabidopsis*^[22−31]^, rice^[32−34]^, maize^[35,36]^, peanut^[37]^, tobacco^[38]^, strawberry^[39]^ and cabbage^[40,41]^, and it has been instrumental in the characterization of distinct cell types in various tissues such as root, stem, leaf, and shoot apex (Fig. 1).

Until 2021, scRNA-seq was applied to the study of woody plants and showed great potential for development. Besides scRNA-seq, emerging technologies, such as single-cell epigenetics and spatial transcriptomics, have introduced new avenues for unraveling the mysteries that surround woody plants. This article offers an in-depth review of the development and data analysis pipeline of scRNA-seq, its applications and challenges in woody plant research, and the potential applications of single-cell epigenetics and spatial transcriptomics.

## Development of scRNA-seq technologies

**Figure 2:**
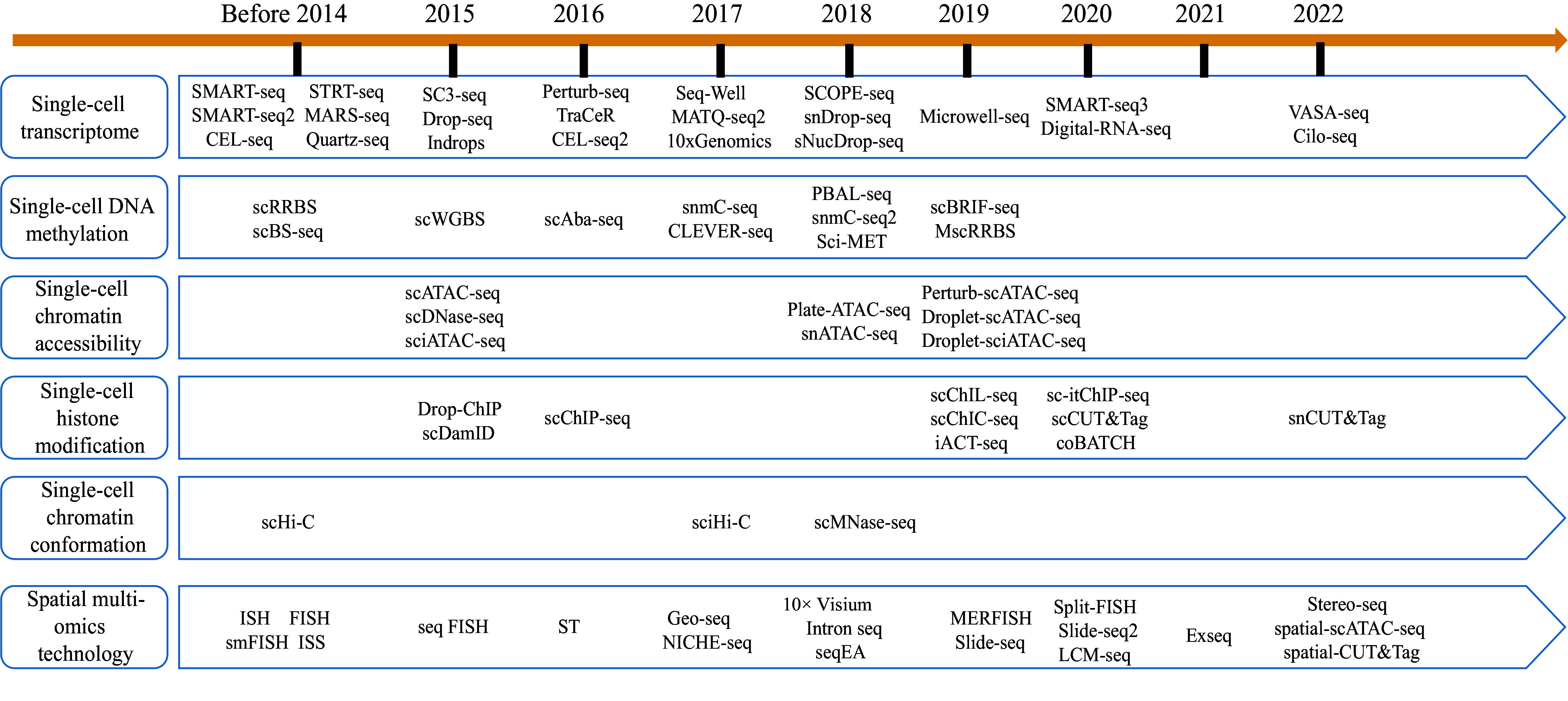
Development history of single-cell sequencing technologies and spatial multiomics technologies.

The fundamental concept of single-cell transcriptome sequencing is the ability to extract RNA from a single cell and to construct a transcriptome library. High-throughput sequencing is then used to read the library, thereby yielding the transcript information of a single cell. The library construction methods can be classified into two categories: full-length transcriptome-based library construction methods and tag-based transcriptome-based library construction methods (Fig. 2).

The full-length transcriptome-based library construction methods are known for their robust gene detection capabilities. In 2009, Tang et al. pioneered a single-cell sequencing method^[42]^, marking the beginning of the single-cell genomics era. The method encompasses five key steps: single-cell isolation and lysis, cDNA first-strand reverse transcription, polyA tail addition at the 3' end, cDNA library amplification, and library amplification. Building upon this foundation, Smart-seq2 and Smart-seq3 were developed. Smart-seq2 enhances enzyme thermostability by introducing betaine into the reverse transcription system, thereby increasing cDNA yield^[43]^. Meanwhile, Smart-seq3 introduces Unique Molecular Identifiers (UMIs) for individual transcripts, significantly enhancing the precision and reliability of gene expression detection^[44]^.

Conversely, the tag-based transcriptome-based library construction method offers higher detection throughput. The principle involves adding primers with cell-specific barcodes during reverse transcription and then using high-throughput sequencing to distinguish transcripts from different cells. Representative technologies utilizing this library construction method include CEL-Seq2, Microwell-seq, MARS-seq, and 10× Genomics Chromium. CEL-Seq2, for example, employs a dual barcode system to identify both cells and individual RNA molecules within the same cell, significantly enhancing sequencing data quality and accuracy^[45]^. Microwell-seq utilizes Microwells, specialized agarose microplates, as the single-cell capture platform. These Microwells are reusable, leading to cost-effective large-scale cell sample processing^[46]^. Chen et al. improved upon this approach and developed Microwell-seq2 by optimizing Microwell utilization and enhancing cell detection sensitivity^[46]^. MARS-seq and MARS-seq2 employ fluorescence-activated cell sorting, reducing the risk of sample contamination^[46,47]^. On the other hand, 10× Genomics Chromium integrates barcoding and microfluidics and uses the Illumina sequencing platform, allowing for the labeling of hundreds of thousands of cells within minutes. Notably, both MARS-seq2 and 10× Genomics Chromium have been applied to woody plant research.

## scRNA-seq data analysis pipeline

**Figure 3:**
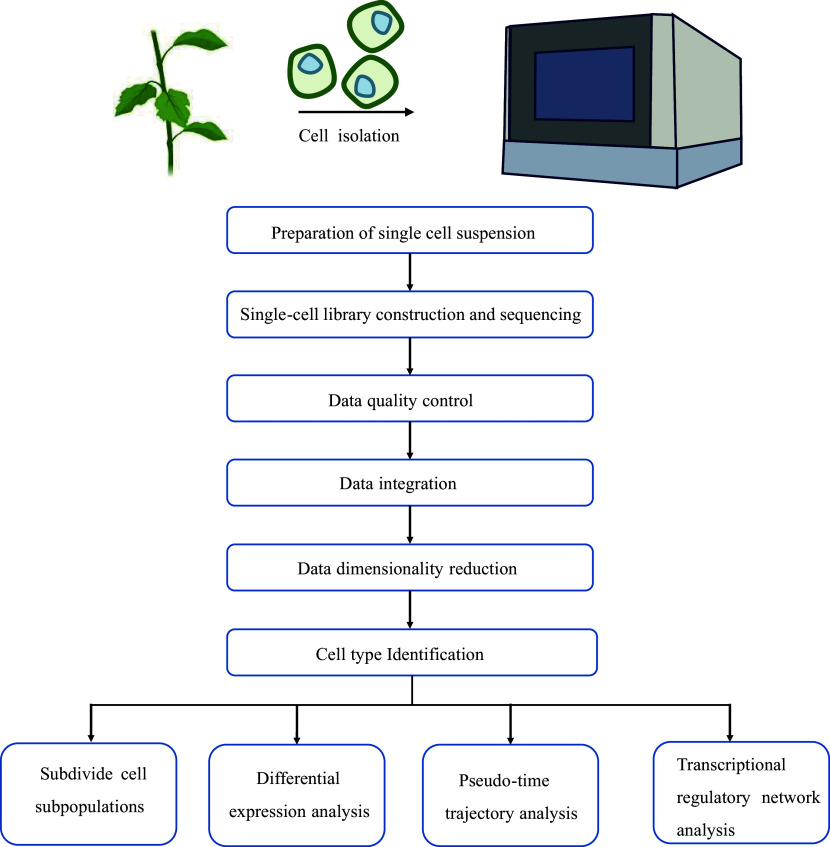
scRNA-seq data analysis pipeline.

Currently, woody plant research mainly utilizes the 10× Genomics Chromium platform. Therefore, we present an overview of the scRNA-seq data analysis workflow based on the 10× Genomics Chromium platform, which includes data quality control, data integration, data dimensionality reduction, cell type identification, and pseudo-time trajectory analysis (Fig. 3).

### Data quality control

To ensure the reliability of the subsequent data analysis, it is necessary to first perform a quality control step on the single-cell data. To achieve this, Seurat^[48]^ is employed to eliminate low-quality cells and genes, while software options such as Doubletfinder^[49]^, Scrublet^[50]^, and DoubletDecon^[51]^ are effective in removing double cells. Among these tools, Doubletfinder stands out as one of the most accurate for doublet removal^[52]^. Doubletfinder initially calculates the proportion of artificial nearest neighbors (pANN) within the nearest neighbor of each individual cell. It then assigns a probability score for doublets to each barcode based on the pANN value. Finally, using the Poisson distribution, Doubletfinder calculates the number of doublets present in each sample and efficiently filters out the doublets, all while taking into account the prior cell pANN value ranking.

### Data integration

For comprehensive analyses and comparisons, it is often necessary to integrate data from different samples or experimental batches, which can be accomplished by software such as Harmony^[53]^, LIGER^[54]^, scMerge^[55]^, scGen^[56]^, and Seurat3^[48]^. Among these options, Harmony stands out for its efficiency when handling large volumes of cellular data, while LIGER excels when the integrated samples show highly variable cell types^[57]^. Other popular methods for data integration and batch effects removal include mutual nearest neighbors (MNN)^[58]^ and canonical correlation analysis (CCA)^[59]^, both of which are available in Seurat3^[48]^. However, investigators should be cautious of overcorrection issues when using these methods. Following data integration, it is crucial to consider the distribution of cell cycle genes. If their distribution is uneven, their effects need to be eliminated, and this can be achieved through the SCTransform function in Seurat3^[48]^.

### Data dimensionality reduction

scRNA-seq data presents a complex high-dimensional structure, involving a multitude of cells and genes, making it challenging to visualize the data in its raw form. Therefore, the data must be dimensionally reduced. Principal Component Analysis (PCA)^[60]^ is the primary method for dimensionality reduction in single-cell data, and it can be further complemented by techniques such as t-Distributed Stochastic Neighbor Embedding (tSNE)^[61]^ and Uniform Manifold Approximation and Projection (UMAP)^[62]^ for data visualization. After the dimensionality reduction of data, spectral clustering based on shared nearest neighbor (SNN) and modular optimization of Seurat can be applied to identify the cell clusters.

### Cell type identification

Cell type identification is a critical step in the scRNA-seq analysis pipeline. In single-cell studies of *Arabidopsis*, the identification of cell types often relies on previously experimentally validated marker genes. *Arabidopsis* has many reliable marker genes such as the endodermal marker gene *MYB36*^[63]^ in roots and the mesophyll marker gene *CAB3*^[64]^ in leaves. However, given the limited availability of marker genes in woody plants, a common strategy is to leverage homologous genes from *Arabidopsis* as references for cell type annotations based on functional conservation. Additionally, *in situ* hybridization and laser capture microdissection (LCM) are candidate methods that can be used to validate the annotation of cell populations.

### Pseudo-time trajectory analysis

To understand the mechanism of organ formation in woody plants, it is important to comprehend the developmental trajectories of various cell types. Pseudo-time trajectory analysis reshapes the change process of cells over time by constructing the transition between cells. Common methods for pseudo-time trajectory analysis include Monocle DDRTree (Monocle2)^[65]^, Slingshot^[66]^, and pCreode^[67]^. Among these options, Monocle2 is frequently used to construct organ developmental trajectories in woody plants^[68−74]^. Monocle2 utilizes a reverse graph embedding machine learning technique to construct cell developmental trajectories. Often, there are multiple branch points in the results of pseudo-time trajectory analysis, and these branch nodes represent cell state changes, so they are of great importance in the analysis of branch events. The BEAM (Branch Expression Analysis Modeling) function in Monocle2 is used to analyze the differential expression of genes at specified nodes, which can play important roles in cell development. However, the cell differentiation trajectory analyzed by Monocle2 is separated from the results of UMAP or tSNE that obtained by dimensionality reduction. Furthermore, when dealing with a large amount of cell data, Monocle2 may cluster cells with different developmental trajectories into the same trajectory. To address this issue, Cao et al. developed Monocle3 based on the partition-based graph abstraction (PAGA)^[75]^, which can directly draw cell development trajectories on UMAP and efficiently analyze millions of single-cell data^[76]^.

In addition to pseudo-time trajectory analysis, the prediction of potential cell fate can also be achieved through RNA velocity analysis. This approach leverages splicing information to determine the directionality of cell differentiation^[77]^. Unlike pseudo-time trajectory analysis, RNA velocity analysis does not yield a continuous cell trajectory but instead provides insights into potential directions in cell differentiation, so there is no need to rely on prior biological experience to specify the start and end points in the analysis. Software tools, such as scVelo^[78]^, Velocyto^[77]^, and PhyloVelo^[79]^, can be used for RNA velocity analysis and combined with pseudo-time trajectory analysis to infer the complex developmental trajectory of woody plant cells.

## Application of scRNA-seq in woody plant research

### Vascular tissue of woody plants

Poplar, renowned for its high genetic transformation and rapid growth, has long served as a model system in woody plant research^[80−82]^. Several species of *Populus* have been sequenced, laying a solid foundation for in-depth research^[83−86]^. Additionally, given its economic value as a tree species, poplar is a prime source of high-quality wood. However, enhancing wood properties requires a comprehensive understanding of the various processes that underlie cell formation and differentiation during wood formation, which are challenging to monitor through traditional molecular biology techniques. Thus, the initial application of scRNA-seq in woody plants has centered around wood development in poplar.

**Figure 4:**
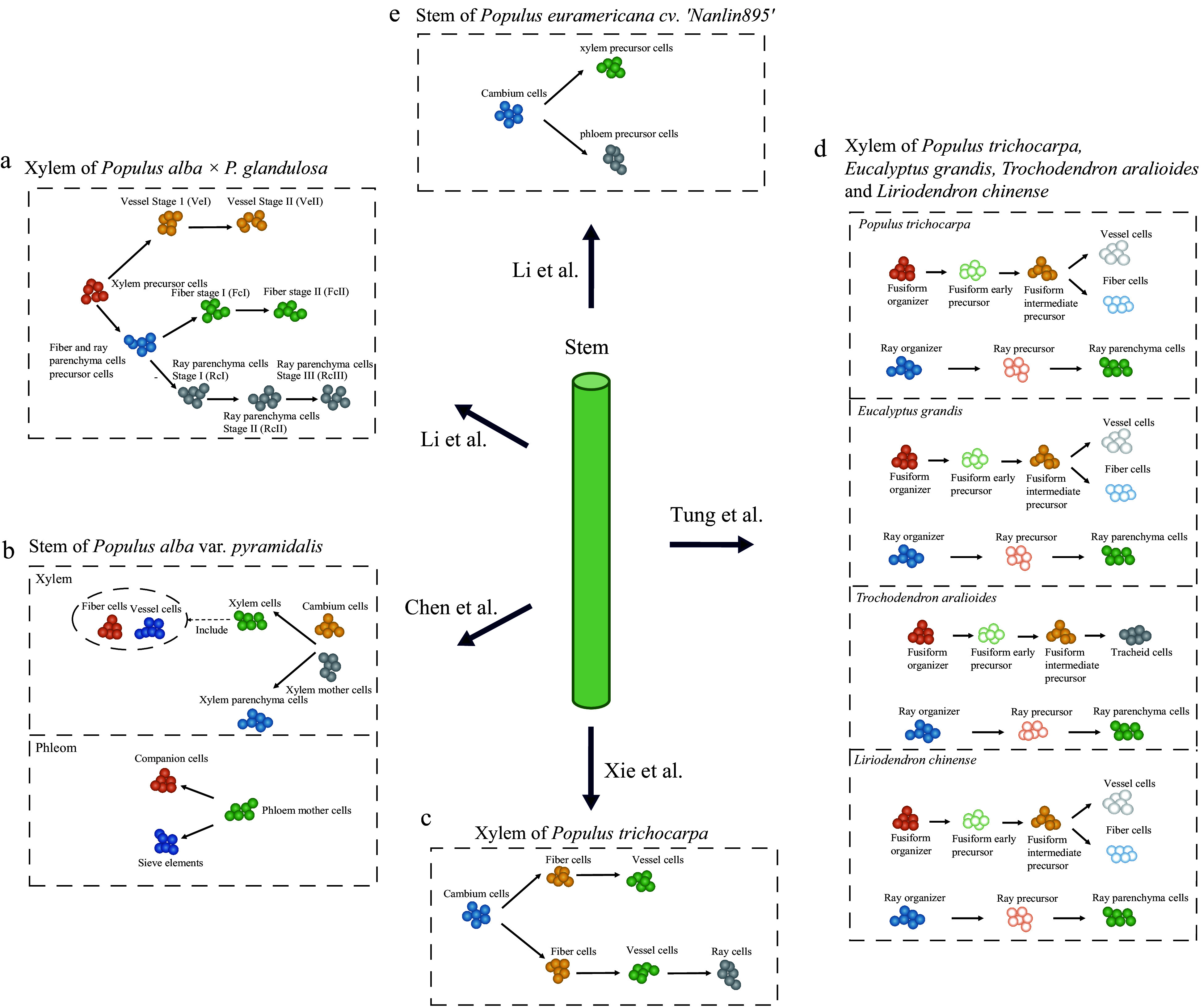
scRNA-seq studies of poplar stem. The balls of different colors represent different types of cells and the arrows point to the direction of cell differentiation.

According to an anatomical identification study, the xylem is primarily comprised of three cell types: fiber, vessel element, and ray^[87]^. As mentioned earlier, the characterization of cell types in most non-model species often relies on homologs of marker genes from *Arabidopsis*. While several molecular markers can be used to identify fiber and vessel elements, it is difficult to identify molecular markers for ray cells because they do not exist in *Arabidopsis*^[88]^. To our knowledge, five studies have recently used scRNA-seq to conduct preliminary analyses of cell types and growth dynamics in poplar woody tissues^[68−70,89,90]^. Among these, Li et al.^[68,90]^ and Chen et al.^[69]^ used molecular markers derived from both poplar and *Arabidopsis*, respectively, while most of the cellular annotations of Xie et al. were based on molecular markers specific to poplar^[70]^. Tung et al. generated *in situ* cell transcriptomes using LCM and separated fibers, vessel elements, and ray cells by ranking the expression correlations between *in situ* cell transcriptomes and cell clusters^[89]^. Because the poplar species used in these studies were different, we integrated these gene markers for cell identity through their orthologous relationships (Supplemental Table S1). Unexpectedly, the results showed that the markers varied widely among these studies. For example, among the molecular markers used by Xie et al, only *IRX1* (xylem cells marker) and *AIL5* (cambium region) were found in other studies^[70]^, while Tung et al. inferred only one gene encoding expansin (*Potri.001G240900*) as a candidate marker for identifying vessel elements^[89]^. Moreover, these studies also identified other cell types in the developing xylem, such as xylem mother cells, organizer cells, and xylem precursor cells, besides the three known types. These cells may be in transitional stages of their developmental fate, and their transcription levels may differ from those of the known cell types. Nevertheless, these studies consistently indicate that the cellular composition of woody tissue is far more complex than anatomically identified, and the characterization of these cells warrants further investigation. In addition to the differences in cell annotation, four out of the five studies also constructed xylem cell developmental trajectories, which showed variations (Fig. 4). Chen et al.^[69]^ and Tung et al.^[89]^ both suggested that fibers and vessel elements belong to the same lineage (Fig. 4b, d), while Li et al. showed that fibers and ray cells shared a common developmental trajectory branch^[68]^ (Fig. 4a). In comparison, Xie et al. constructed two trajectories of cellular differentiation, with one differentiating into fibers and vessel elements and the other into fibers, vessel elements, and ray cells^[70]^ (Fig. 4c). Notably, these differences in cell differentiation trajectories may be influenced by the identification of cell types.

Furthermore, Chen et al. and Li et al. performed analyses of phloem cells. In both studies, *CalS7* and *SEOR1* were used to identify sieve elements. These investigators also identified the phloem precursor cells (phloem mother cells), cortex cells, epidermal cells, and companion cells. Chen et al. also identified photosynthetic cells, phloem parenchyma cells, cork cells, and endodermal cells, as well as reconstructed the cell differentiation trajectories of the phloem with phloem mother cells, companion cells, and sieve elements^[69]^, while Li et al. reconstructed the cell differentiation trajectories of the cambium to the phloem precursor and xylem precursor^[90]^.

In addition to their investigations on poplar xylem and phloem cell development, Chen et al. further used single-cell transcriptome data to predict potential gene redundancy resulting from whole-genome duplication (WGD) in Salicaceae^[69]^. Meanwhile, Tung et al. identified highly conserved ray lineages and variable fusiform lineages based on comparative single-cell mapping of four different woody angiosperms (*Populus trichocarpa*, *Eucalyptus grandis*, *Trochodendron aralioides*, and *Liriodendron chinense*)^[89]^. In summary, these studies underscore the high degree of heterogeneity within cell populations in wood, a phenomenon not previously observed in anatomical studies. These studies also highlight the importance of single-cell transcriptomics in the study of woody plants. The discrepancy between these studies may be attributed to the dynamic nature of cell development, where the data only shows a snapshot of the entire developmental process. Additionally, differences in growth conditions, experimental conditions, and expression variations between species may also lead to differences in cellular annotations and developmental trajectories. However, because different marker genes were used, it is difficult to assess whether these differences are due to artifacts in cell identification. One promising approach is to integrate these data and use the same criteria to characterize their cell types and perform comparative studies.

### Other tissues of woody plants

In addition to the various studies conducted on different poplar species, scRNA-seq has also been applied to other woody plants. For example, four reports have used scRNA-seq to study cotton, two of which identified the cell types in the ovule’s outer layer and the genes related to cotton fiber synthesis, while the other two focused on the cell types within the cotyledons and the genes affecting the formation of pigment glands^[91−94]^ (Table 1). Ding et al. analyzed the initiation process of *Bombax ceiba* fiber and compared it with single-cell mapping of cotton fiber to excavate genes associated with fiber development^[95]^ (Table 1). *Camellia sinensis*, one of the most important woody crops cultivated globally, is known for its leaves, which produce various teas^[96]^. Wang et al. analyzed the spatiotemporal expression patterns of flavor-related genes in different cell types of *C. sinensis* leaves and discovered a new catechin glycosyltransferase (UGT72B23)^[71]^ (Table 1). On the other hand, *Hevea brasiliensis*, an important source of natural rubber^[97]^, was investigated by Liang et al., who constructed single-cell maps of normal and powdery mildew-inoculated *H. brasiliensis* leaves and identified a powdery mildew resistance gene *HbCNL2* through pseudo-time trajectory and phylogenetic analyses. Its function was also verified by molecular biology methods^[72]^ (Table 1). Taxol, a highly effective anticancer drug, is a component of *Taxus chinensis*^[98]^, and Zhan et al. used scRNA-seq and mass spectrometry to reveal the molecular mechanism of the cell-specific secondary metabolism in *T. chinensis* leaves^[74]^ (Table 1). In summary, these studies showed that scRNA-seq opens new avenues for us to explore the mysteries of woody plants.

**Table 1:** Application of scRNA-seq/snRNA-seq technologies in woody plants.

Species	Tissue	Cell type	Sequencing method/ platform	Reference
*Populus alba* × *Populus glandulosa*	Xylem	Xylem precursor cells, fiber cells, vessels cells, and ray parenchyma cells	10× Genomics	[68]
*Populus alba* var. *pyramidalis*	Stem	Photosynthetic cells, cambium cells, phloem parenchyma cells, xylem cells, xylem mother cells, phloem mother cells, endodermal cells, xylem parenchyma cells, cork cells, epidermal cells, companion cells, cortex/endodermal cells, cortex/endodermis initial cells, and sieve elements	10× Genomics	[69]
*Populus trichocarpa*	Xylem	Vessel cells, ray parenchyma cells, phloem cells, cambium cells, and fiber cells	10× Genomics	[70]
*Populus trichocarpa*	Xylem	Fusiform organizer, fusiform early precursor, fusiform intermediate precursor, vessel cells, fiber cells, ray organizer, ray precursor, and ray parenchyma cells	10× Genomics and MARS-seq2.0	[89]
*Eucalyptus grandis*	Xylem	Fusiform organizer, fusiform early precursor, fusiform intermediate precursor, vessel cells, fiber cells, ray organizer, ray precursor, and ray parenchyma cells		
*Trochodendron aralioides*	Xylem	Fusiform organizer, fusiform early precursor, fusiform intermediate precursor, tracheid cells, ray organizer, ray precursor, and ray parenchyma cells		
*Liriodendron chinense*	Xylem	Fusiform organizer, fusiform early precursor, fusiform intermediate precursor, vessel cells, fiber cells, ray organizer, ray precursor, and ray parenchyma cells		
*Populus euramerican*a cv. ‘Nanlin895’	Stem	Phloem precursor, xylem precursor, cambium, vessel, cortex and pith, ray, epidermis, sieve-companion, xylem fiber, and phloem parenchyma	10× Genomics	[90]
*Camellia sinensis*	Leaf	Vascular bundle, protoxylem cells, protophloem cells, phloem cells, procambium cells, proliferating cells, epidermis cells, mesophyll cells, palisade mesophyll, and spongy mesophyll	10× Genomics	[71]
Reyan73397(*Hevea brasiliensis*)	Leaf	Meristem cells, latex cells, xylem cells, phloem cells, hydathode cells, bundle sheath cells, epidermis cells, and mesophyll cells	10× Genomics	[72]
*Bombax ceiba*	Inner wall of the ovary	Initiated fiber cells and epidermal cells in the inner wall of the ovary	10× Genomics	[95]
*Populus tremula × alba*	Shoot apex vascular system	Trichomes, mesophyll cells, epidermal cells, shoot meristematic cells, proliferating cells, vascular cells, companion cells, and ground meristem cells	10× Genomics	[101]
*Taxus mairei*	Stem	Xylem parenchyma cells, xylem cells, epidermal cells, photosynthetic cells, vascular cells, xylem mother cells, companion cells, phloem cells, endodermal cells, cambium cells, and sieve elements	10× Genomics	[73]
*Taxus mairei*	Leaf	Bundle sheath cells, mesophyll cells, stomatal complex cells, guard cells, epidermal cells, vascular cells, procambium cells, and pavement cells	10× Genomics	[74]
The cotton Lint-Fuzz (Xu142_LF)	Ovule outer integument	Fiber, epidermis and outer pigment layer	10× Genomics	[91]
*G. hirsutum cv.* *Xuzhou 142*	Ovule outer integument	Fiber and epidermis	10× Genomics	[92]
Gland cotton 'CCRI12' and glandless cotton 'CCRI12gl'	Cytoledon	Spongy mesophyll cells, palisade mesophyll cells, epidermal cells, primordial cells, guard cells, xylem cells, parenchyma cells, phloem cells, pigment gland cells	10× Genomics	[94]
*Gossypium bickii*	Cytoledon	Mesophyll cells, pigment gland cells, epidermal cells, guard cells, xylem cells, procambium cells, phloem parenchyma cells, and companion cells	10× Genomics	[93]

## Challenges of scRNA-seq in woody plant research

While scRNA-seq has proven successful in woody plant research, it still faces several challenges, including cell separation, cell type annotation, and data integration. First, there are great difficulties in the preparation of woody plant protoplasts due to high lignification and active secondary metabolism, which may render some cells with thick cell walls unusable for analysis. This challenge is clearly evident in recent studies. For example, Li et al. failed to separate the protoplasts of phloem^[68]^, whereas Chen et al. failed to detect cell types or state with strong expression of programmed cell death-related genes^[69]^. To circumvent these difficulties, single-nucleus transcriptome sequencing (snRNA-seq) can be used to isolate cell nuclei. While the gene detection capability of snRNA-seq may not be as robust as that of scRNA-seq, it excels at capturing cells that are not easily digested by enzymes. For example, Guillotin et al. identified columella cells in maize roots by snRNA-seq^[99]^, a feat that had not been achieved in previous studies^[35]^. Recently, Conde et al. developed a nucleus isolation method suitable for tissues with thick secondary cell walls^[100]^ and applied it to the study of shoot apex differentiation in *Populus tremula×alba*. Using snRNA-seq, they identified highly heterogeneous cell populations and performed comparative analyses of vascular development between *Arabidopsis* and poplar (Table 1)^[101]^. This approach not only helped in studying the transition from primary growth to secondary growth in perennial woody plants but also helped in establishing the foundation for the broad application of snRNA-seq to woody plants. These studies illustrate that snRNA-seq can mitigate transcriptome bias during protoplast preparation, thereby enabling the construction a more comprehensive single-cell atlas.

Secondly, the annotation of cell types is equally challenging. The functions of homologous genes are not conserved across species, and this can significantly impact the accuracy of cell annotation. For example, prior studies identified *WOX4* and *PXY* as marker genes of the cambium of *Arabidopsis*^[102,103]^, and these genes were also reported in poplar^[104,105]^. However, Li et al. demonstrated that these two genes are also expressed in other cell types, indicating that they are not reliable molecular markers^[90]^. In this review, we compiled marker genes used in woody plant research. The results showed a wide variation in marker genes between different plant species, as well as between different tissues and even within the same tissue (Supplemental Table S2). Additionally, the manual annotation of cell types is both time-consuming and inefficient, so there is a pressing need to develop an automated process for identifying plant cell types. To date, the PsctH^[106]^, PCMDB^[107]^, and PlantscRNAdb^[108]^ databases have provided us with a wealth of plant marker genes, but the number of marker genes available for woody plants remains limited. Liu et al. proposed a potential design process for creating automated annotation software for plants^[109]^, which provides a theoretical basis for future software development. It is believed that with the mining of conserved marker genes, the establishment of marker gene databases, and the development of automated annotation software, cell type annotation in woody plants will become efficient and accurate.

Thirdly, performing comparative transcriptome analyses provides invaluable insights into the evolutionary and developmental relationships between cell or tissue types of different species. The development of single-cell transcriptomic technologies has opened up new possibilities for investigating cell type phylogenies and inferring cell type-specific evolution. Comparative analyses of cell types require quantifying the similarity in gene expression profiles, which often relies on gene homologous relationships between species. Several tools, such as SAMap^[110]^ and CAME^[111]^, have been developed and are mostly used in animal studies where orthologous relationships are relatively clear. However, plants often undergone independent genome duplications^[112,113]^, the complex genetic relationships make the integration of cross-species data extremely difficult. For example, Conde et al. integrated *Arabidopsis* and poplar data using a one-to-one orthologous gene approach^[101]^, while Tung et al. employed many-to-many homologous clusters for cross-species integration and comparison^[89]^. It is important to note that the one-to-one approach introduces complexity when integrating distantly related species because there are fewer orthologous genes, and differences in WGD-derived duplicates may confound orthologous relationships. In contrast, the many-to-many approach may mask functional divergence and neofunctionalization of paralogous genes, making it difficult to accurately assess their diversification history. Comparative studies in plants are therefore relatively limited. A potential solution is thus to establish comparison methods that do not rely on homologous genes. For example, Random Forest Machine Learning (RFML) trains algorithms against the cell types of one species and then predicts interspecies cell type similarity^[114]^. This approach has been successfully applied in studies involving animals^[115,116]^. However, further studies are needed in the future to determine the best methods for comparing single-cell transcriptomes between plant species.

## Application prospects of single-cell epigenetics technologies in woody plants

Heterogeneity in cellular transcriptional expression is often determined by heterogeneity in epigenetic modifications. Similar to transcriptomics, traditional epigenetic techniques examine entire tissues but overlook cellular heterogeneity^[117]^. In recent years, various single-cell sequencing technologies have emerged, each addressing different levels of epigenetic regulation (Fig. 2). These techniques mainly employ different enzymes to process chromatin and capture target information. Some techniques have been applied to plant research. For example, scBRIF-seq enables the study of DNA methylation in single cells. Its pipeline involves single-stranded ligation of small fragments generated through random amplification, MDA amplification, and Tn5-based library construction. This technique has been applied to investigate maize microspores, and significant methylation reprogramming and cellular heterogeneity during maize male gametophyte development were revealed^[118]^. For single-cell chromatin accessibility studies, two primary techniques have evolved: scATAC-seq and snATAC-seq^[119−121]^. ATAC-seq, which assesses genome-wide chromatin accessibility by cutting DNA sequences using Tn5 transposase as a probe, has proven useful for identifying dynamic chromatin changes in response to stress and during the development of woody plants^[122−124]^. ATAC-seq was subsequently modified by combining it with single-cell library construction methods to develop scATAC-seq and snATAC-seq. In plant research, these two methods are often paired with scRNA-seq or snRNA-seq to enable a more in-depth exploration of cellular heterogeneity^[125−127]^. For example, Farmar et al. used snRNA-seq and snATAC-seq to reveal the impact of chromatin accessibility on gene expression in *Arabidopsis* root^[126]^. Wang et al. combined scATAC-seq and scRNA-seq to propose a model for the rhythmic regulation of early cotton fiber growth^[92]^. In terms of single-cell histone modifications, Ouyang et al. developed snCUT&Tag and applied it to the analysis of the characteristics of single-cell H3K4me3 histone modifications in rice seedlings^[128]^. This technology combines nCUT&Tag and single-cell barcode labeling, and its subsequent library construction bear similarity to ATAC-seq. Lastly, single-cell high-throughput chromosome conformation capture (scHi-C) is an important method for analyzing chromatin conformation at the single-cell level^[129]^. Zhou et al. developed the scHi-C technology, which is suitable for plant research, and revealed the changes in the chromatin spatial structure of rice gametes before and after fertilization at the single-cell level^[130]^.

While a variety of single-cell epigenomic technologies have been successfully employed in plant research, their application has been restricted to *Arabidopsis* and certain key crops such as rice and maize. Similar research involving woody plants is very limited. On the one hand, challenges in cell separation and sample preparation for woody plants persist. On the other hand, some technologies lack established analysis pipelines, and eliminating technical noise and batch effects are complex tasks. Therefore, the full potential of these technologies in woody plants has yet to be realized. However, as technologies and software tools continue to advance, there is promise that single-cell epigenetics will offer invaluable insights into the epigenetic landscape of woody plants.

## Application prospects of spatial transcriptomics in woody plants

One of the disadvantages of single-cell transcriptomics is that it destroys the spatial location information of tissues. Spatial transcriptome technologies have emerged to address this limitation, enabling the precise location of various cell types within plant tissues and facilitating the mapping of gene expression across different tissue regions. The current spatial transcriptome technologies primarily fall into four categories: laser microdissection, fluorescence *in situ* hybridization, fluorescence *in situ* sequencing, and *in situ* capture technology (Fig. 2).

In recent years, researchers have combined laser microdissection and next-generation sequencing to develop spatial transcriptome technologies. For example, LCM-seq and Geo-seq can acquire cell transcriptome information while preserving the original location information of cells^[131]^. However, these technologies are time-consuming, and the cell separation process can increase the risk of cell damage. Fluorescence *in situ* hybridization (FISH) offers an alternative approach that uses fluorescently-labeled probes to quantify the abundance of RNA/DNA in cells or tissues without destroying cell morphology, and it has evolved to achieve single-molecule resolution, including sm FISH, seq FISH^[132]^, and intron seq FISH^[133,134]^. While the detection, throughput, and accuracy of these technologies continue to improve, they still face challenges related to complex steps and high costs. Additionally, researchers have developed spatial transcriptome technologies with higher spatial resolution, including ISS^[135]^, FISSEQ^[136,137]^, and Exseq^[138]^, which utilize fluorescently-labeled probes that hybridize with target sequences, allowing the determination of the location of the target sequence by observing the location and intensity of the fluorescent signals under a microscope. However, some issues still remain, including short sequencing read length and low detection efficiency.

Compared with the above methods, spatial transcriptomics based on *in situ* capture technology offers several advantages, including the ability to achieve high throughput and large tissue areas. The principle is to capture transcripts *in situ* using primer microarrays with spatial tag sequences, thereby preserving the spatial information of the transcripts. Stahl et al. pioneered spatial transcriptomics^[139]^, and it was rapidly applied to plant research. For example, Giacomello et al. constructed spatial transcriptome maps of the inflorescence meristems of *Arabidopsis thaliana*, the female autumn flowers of *Picea abies*, and the leaf buds of *Populus tremula*^[140]^. More importantly, these studies revealed the suitability of spatial transcriptomics for the study of woody plants. Subsequently, Du et al. used spatial transcriptomics to analyze the continuous development of poplar stems from primary growth to secondary growth, and they identified a new class of procambium-like cells that specifically develop into the phloem^[141]^. Li et al. constructed a spatial transcriptome map of poplar stems and analyzed gene expression in various cell types^[90] ^. These studies demonstrate that spatial transcriptomics opens up new dimensions and avenues for discoveries in woody plant research. In addition, Stereo-seq^[142]^, a new spatial transcriptome technology, was recently used to study *Arabidopsis* leaves^[143]^. This study successfully distinguished upper and lower epidermal cells of leaves and analyzed the expression changes of genes related to photosynthesis from leaf veins to leaf edges. The technique’s advantage lies in its nanoscale resolution and its precise identification of cell subtypes, which presents new opportunities for the study of woody plants. Besides spatial transcriptomics, other spatial multiomics technologies are also progressing rapidly. DBiT-seq realizes the simultaneous acquisition of transcriptomic and proteomic information while acquiring spatial information^[144]^, whereas spatial-ATAC-seq^[145]^ and spatial-CUT&Tag^[146]^ enable the study of chromatin accessibility and histone modifications at spatial resolution. However, these techniques have yet to be applied to plant research.

While spatial transcriptomics has experienced rapid advancements, technical difficulties still hinder its application in woody plant research. Currently, most spatial transcriptome studies are carried out on young tissues characterized by thin cell walls that are easily processed for freezing and embedding. However, tissues with a high degree of lignification may be damaged during processing, potentially compromising the integrity of reverse transcription products and the subsequent analysis. Therefore, future studies should focus on further optimizing the sample preparation protocol. On a more positive note, the subsequent data analysis process continues to mature, and software tools, including Seurat^[48]^, scanpy^[147]^, squidpy^[148]^, STUtility^[149]^, and Giotto^[150]^, can be used for spatial data analysis and visualization. It is believed that with continued technological advancements, spatial transcriptome technologies will offer new perspectives on the growth and development, stress resistance, and species evolution of woody plants.

## Conclusions

**Figure 5:**
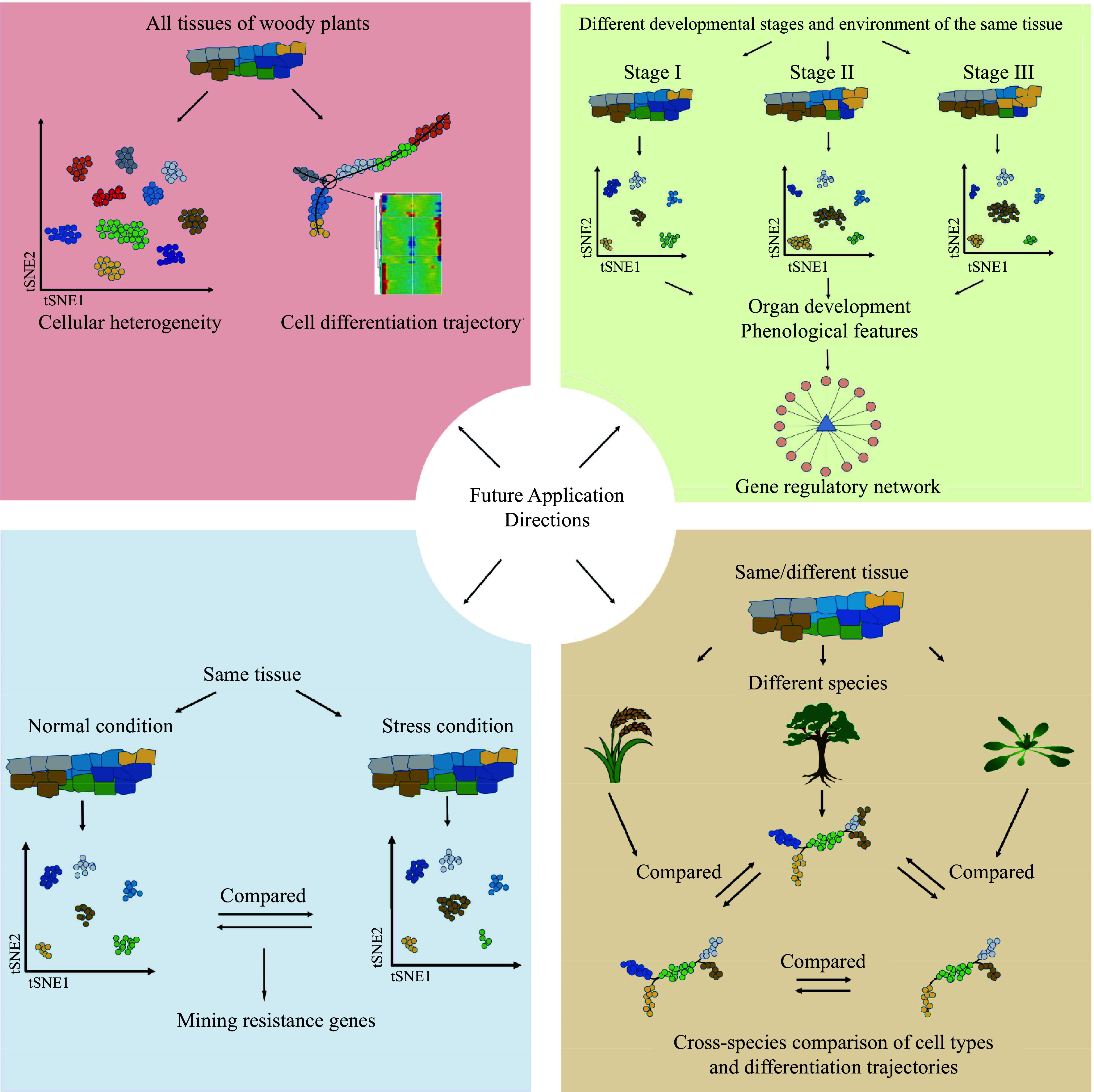
Future application of single-cell transcriptomics, single-cell epigenetics, and spatial transcriptomics in woody plants.

Studies involving single-cell transcriptomics, single-cell epigenetics, and spatial transcriptomics have been limited in woody plants compared to other organisms. Current research primarily focuses on a small number of species and young, fresh tissues. As a consequence, there is a pressing need to expand the application of these techniques to a broader range of woody plant species and diverse tissue types. In this regard, snRNA-seq has emerged as a powerful tool to help achieve these goals. In single-cell research, a comprehensive human pan-tissue single-cell atlas has been successfully established^[151−155]^. Similarly, the creation of pan-tissue single-cell atlases covering different tissues, developmental stages, and environmental conditions in woody plants will significantly enhance our understanding of the diversity of cell types, and provide new perspectives for studying the evolution and origin of specific traits in woody plants. But before that, it will be important to develop and integrate species-specific and conserved molecular markers for precise identification of cell types (such as Supplemental Tables S1 & S2 in this review). However, current studies lack inter- and intra-species comparisons of single-cell atlases. Interspecific comparisons can illuminate the similarities, differences, and evolutionary relationships of cells among different plant phyla and classes. At the same time, intraspecies comparisons can be employed to investigate functional variations among different tissues (Fig. 5). Therefore, it is reasonable to expect that as single-cell and spatial transcriptome techniques become more widely employed, our knowledge of woody plants will significantly advance in the future.

## SUPPLEMENTARY DATA

Supplementary data to this article can be found online.

## Data Availability

Data sharing is not applicable to this article as no datasets were generated or analyzed during the current study.
